# Intestinal microbial metabolite stercobilin involvement in the chronic inflammation of *ob/ob* mice

**DOI:** 10.1038/s41598-020-63627-y

**Published:** 2020-04-15

**Authors:** Shunsuke Sanada, Takuji Suzuki, Akika Nagata, Tsutomu Hashidume, Yuko Yoshikawa, Noriyuki Miyoshi

**Affiliations:** 1 Graduate School of Integrated Pharmaceutical and Nutritional Sciences, University of Shizuoka, Shizuoka, Japan; 2 Food Environmental Design Course, Faculty of Education, Art and Science, Yamagata University, Yamagata, Japan; 3 School of Veterinary Medicine, Faculty of Veterinary Science, Nippon Veterinary and Life Science University, Tokyo, Japan

**Keywords:** Biomarkers, Metabolomics

## Abstract

It is crucial that the host and intestinal microflora interact and influence each other to maintain homeostasis and trigger pathological processes. Recent studies have shown that transplantation of the murine intestinal content to recipient germ-free mice enables transmission of the donor’s phenotypes, such as low level chronic inflammation associated with lifestyle-related diseases. These findings indicate that intestinal bacteria produce some molecules to trigger pathological signals. However, fecal microbial metabolites that induce obesity and the type II diabetic phenotype have not been fully clarified. Here, we showed that the intestinal bacterial metabolite stercobilin, a pigment of feces, induced proinflammatory activities including TNF-α and IL-1β induction in mouse macrophage RAW264 cells. Proinflammatory stercobilin levels were significantly higher in *ob/ob* mice feces than in the feces of control C57BL/6 J mice. Moreover, in this study, we detected stercobilin in mice plasma for the first time, and the levels were higher in *ob/ob* mice than that of C57BL/6 J mice. Therefore, stercobilin is potentially reabsorbed, circulated through the blood system, and contributes to low level chronic inflammation in *ob/ob* mice. Since, stercobilin is a bioactive metabolite, it could be a potentially promising biomarker for diagnosis. Further analyses to elucidate the metabolic rate and the reabsorption mechanism of stercobilin may provide possible therapeutic and preventive targets.

## Introduction

It is becoming clear that the intestinal microflora and host communicate with each other to regulate homeostasis and sometimes trigger pathophysiological changes^1,2^. A pioneering study reported that fecal microbiota transplantation enabled transmission of several phenotypes from the donor, including insulin resistance, fat accumulation, obesity, hyperglycemia, hypertriacylglycerolemia, hypercholesterolemia, hypertension, hyperphagia, and low level chronic inflammation^3,4^. These conditions were negated by the treatment with antibiotics, showing that they originated from fecal microbiota transplantation. However, it is not understood which molecules mediate the transmission of pathophysiological changes from donor to recipient. Additionally, it has been pointed out that the deterioration of intestinal microbiota in humans is related not only to lifestyle-related diseases, including obesity, diabetes, cancer, and cardiovascular diseases, but also to many other diseases, such as dementia^5^ and mental diseases^6^. One reason for this is inflammation, which is a common risk factor. Therefore, moderate or low level chronic inflammation induced by bacterial molecules is thought to be a fundamental mechanism for triggering and promoting these disorders because of interactive communication between host and intestinal bacteria.

In addition to 16 s rRNA sequencing, metabolomics analysis is an effective approach to understanding how the host and bacteria communicate in the intestinal environment. It has been revealed that some bioactive intestinal and microbial metabolites were strongly associated with homeostasis and disease etiology. For example, representative volatile organic compounds in the intestinal tract include short-chain fatty acids (SCFAs). SCFAs produced by intestinal fermentation are consumed as an energy source and function as bioactive metabolites for immune regulation, suppression of food intake, and enhancing the intestinal barrier function, which mediates anti-obesity, anti-diabetes, and anti-carcinogenesis effects^7–12^. Moreover, deoxycholate generated by the metabolism of intestinal microbiota induces DNA damage and inflammation, which might play a pivotal role in obesity-associated hepatic carcinogenesis^13^. Another example is the metabolites of dietary lipid phosphatidylcholine (lecithin), including trimethylamine *N*-oxide (TMAO) and betaine, whose levels are strongly associated with the pathogenesis of cardiovascular diseases^14,15^. Of note, TMAO and betaine have proinflammatory activities that induce scavenger receptors on macrophages. Similar to compounds such as deoxycholate, TMAO and betaine, bioactive metabolites have advantages as predictive biomarkers. These “bioactive” biomarkers trigger, initiate, and promote pathological events, and therefore might be useful in the diagnosis in early stage diseases.

Urobilinoids are a naturally occurring bile pigment formed by intestinal bacteria in the heme catabolic pathway^16^. Heme released from hemoglobin during several pathophysiological states is sequentially converted to biliverdin by hemeoxygenase, then to bilirubin by biliverdin reductase^17^. Bilirubin diglucuronide formed by uridine diphosphate glucuronosyl transferase (UGT) is secreted from hepatocytes to bile via the MRP2 transporter^18^. Bilirubin diglucuronide is deconjugated and converted by intestinal bacteria to form urobilinoids, which are a group of metabolites consisting of porphyrin derivative. The four major urobilinoids are urobilinogen, urobilin, stercobilinogen, and stercobilin. Among them, urobilinogen is a major primary urobilinoid, some of which is absorbed in the intestine and transported to the kidney. Then, urobilinogen and its oxidized form, urobilin, are excreted in urine. Another part of urobilinogen is enzymatically reduced by intestinal bacteria to form stercobilinogen. Then, stercobilinogen and its oxidized form, stercobilin, are excreted in feces^16^. Indeed, some hepatopathies cause pathologically elevated levels of plasma bilirubin. However, the physiological level of bilirubin seems to be beneficial for preventing obesity and diabetes because bilirubin directly binds with peroxisome proliferator-activated receptor alpha (PPARα), and positively drive the fatty acid β-oxidation^19^. However, although urobilinoids are used to monitor fecal pollution^20^, few studies addressing the bioactivities in the human body have been published previously.

**Figure 1:**
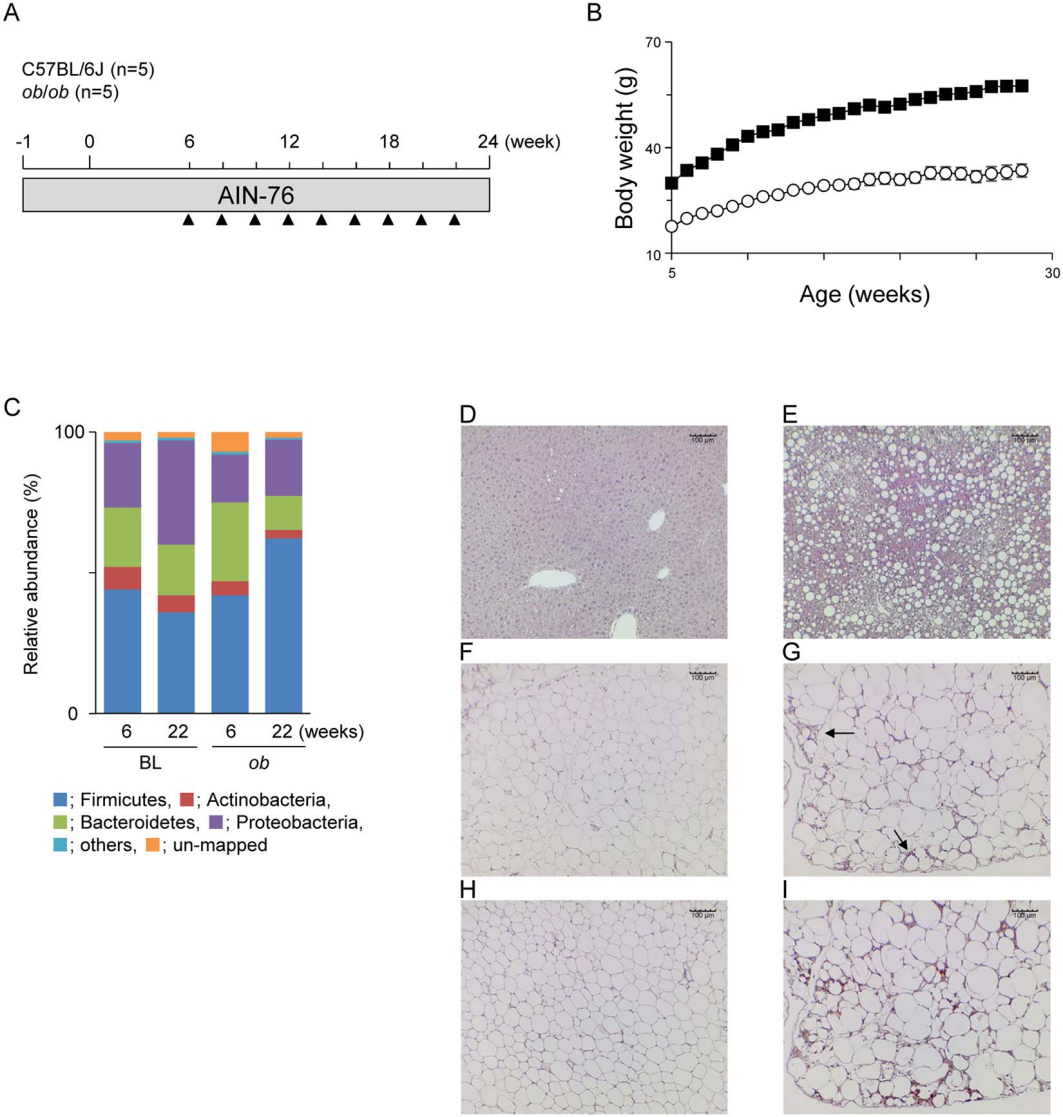
Experimental protocols and pathophysiology of C57BL/6 J and *ob*/*ob* mice. (**A**) Experimental protocol used in this study. C57BL/6 J mice (n = 5) and *ob*/*ob* mice (n = 5) were fed a AIN-76 diet until the end of the experimental period. Fecal samples were collected at the indicated time points (▲). (**B**) Body weight changes of C57BL/6 J (○) and *ob*/*ob* mice (■) during the experimental period. Data are presented as mean ± SEM. (**C**) Microbial composition in mice feces. 16 s rRNA sequencing was performed. Relative abundance and taxonomic classification at the phylum level were analyzed by Ion Reporter. HE staining of liver (**D**,**E**) and white adipose tissues (WAT) (**F**,**G**), and immunohistochemistry for F4/80 in WAT (H and I). Mouse tissues collected from C57BL/6 J (D and F) and *ob*/*ob* mice (**E** and **G**) at week 24 were prepared and stained with HE. Representative images (scale bar = 100 µm) are shown. Arrows indicate infiltrated macrophages (**G**).

In this study, we performed metabolomics analyses to determine proinflammatory metabolites in the feces of obese/diabetic model *ob*/*ob* mice (Fig. 1A). We found that stercobilin, a urobilinoid, induced proinflammatory activities and was higher in *ob/ob* feces and plasma compared with those of C57BL/6 J mice. These findings suggest that an elevated level of fecal stercobilin is potentially reabsorbed, systemically circulated in the body, and contributes to low level chronic inflammation in *ob/ob* mice.

## Results

### Pathological features of ob/ob mice

**Figure 2:**
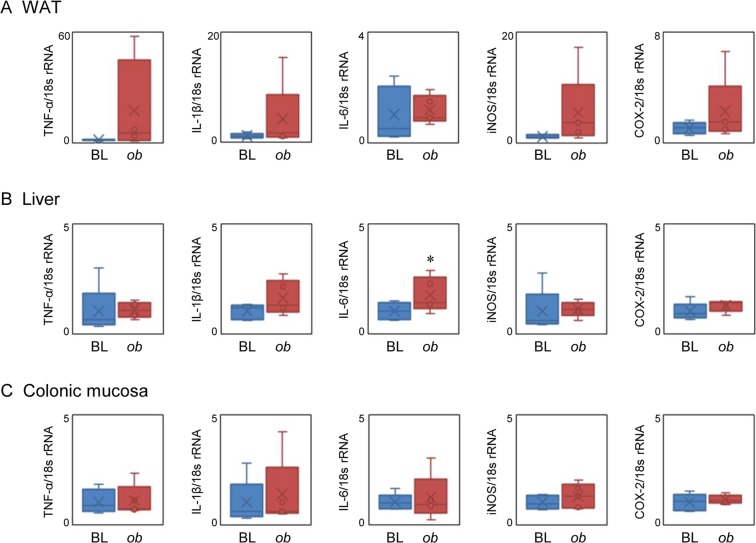
Inflammatory gene expression in C57BL/6 J (BL) and *ob/ob* (*ob*) mice white adipose tissue (WAT) (**A**), liver (**B**), colonic mucosa (**C**). RT-qPCR was performed as described in the Materials and Methods. Data are shown by whisker plot (n = 4~5). *p < 0.05 compared with BL (*t*-test).

The body weight of *ob*/*ob* mice was significantly higher than that of C57BL6/J mice for all experimental periods (Fig. 1B). The fecal 16 s rRNA sequence in feces collected at weeks 6 and 22 demonstrated that, as reported by other studies, the number of *Firmicutes* in *ob*/*ob* mice increased with a corresponding decrease in *Bacteroidetes* in an age-dependent manner (Fig. 1C). Five bacterial family/genus were statistically significance by two-way ANOVA in week or mouse lineage (Table S1). Especially, the ratio of *Lachnospiraceae*, one of the bacteria of *Firmicutes*, was markedly higher in *ob/ob* mice (Table S1). Several fatty liver observations, such as hepatic ballooning degradation and fat droplets, were found in *ob*/*ob* mice (Fig. 1D,E). Additionally, adipocyte hypertrophy (Fig. 1F,G) and macrophage infiltration (Fig. 1H,I) were observed in white adipose tissues of *ob*/*ob* mice, which was strongly associated with the low-level chronic inflammation. mRNA levels of TNF-α and IL-1β in the WAT of *ob*/*ob* mice were, respectively, 17- and 4-fold higher than those of C57BL/6 J mice (Fig. 2A). The iNOS and COX-2 in WAT were also, respectively, 5.4- and 2.2-fold higher than those of C57BL/6 J mice (Fig. 2A). Hepatic gene expression of IL-6 was significantly increased in *ob*/*ob* mice compared with C57BL/6 J mice although TNF-α and IL-1β were not markedly increased (Fig. 2B). In contrast, no obvious elevation of inflammatory genes was observed in the colonic mucosa (Fig. 2C). These results suggest that the microbial composition changes and systemic low-level chronic inflammation were induced in *ob*/*ob* mice.

### Proinflammatory activities of mice fecal aqueous and organic extracts

**Figure 3:**
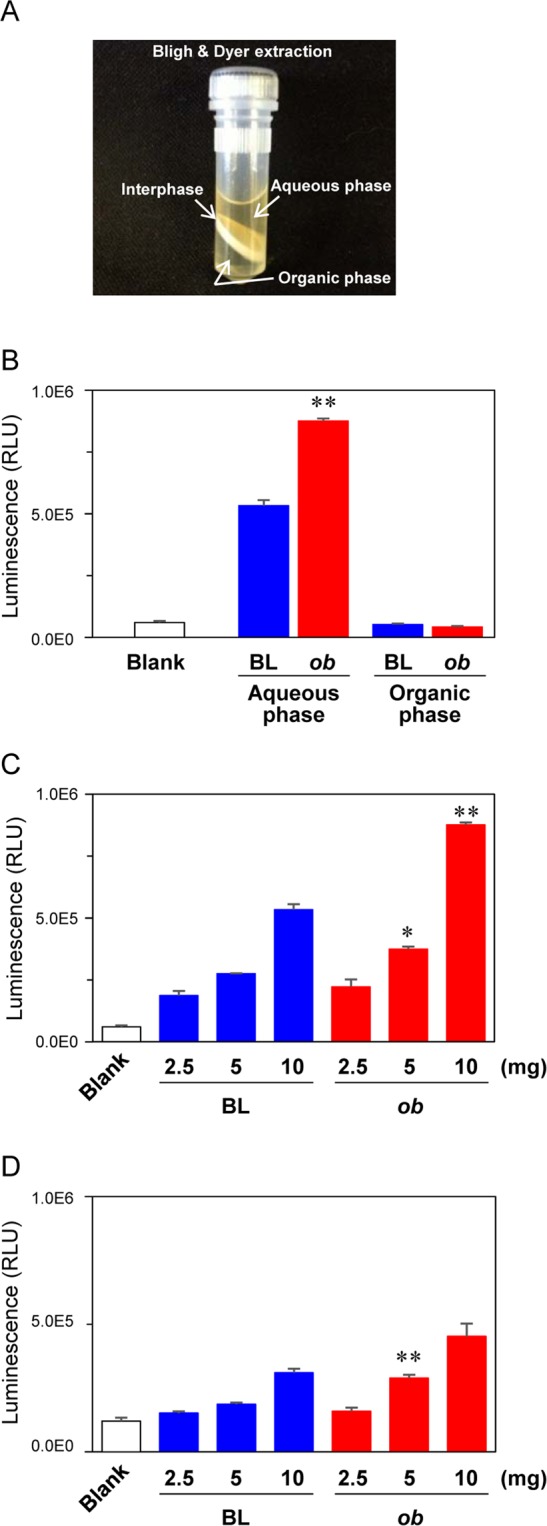
NF-κB reporter gene assay for mice fecal extracts. (**A**) Appearance of Bligh–Dyer extraction of mouse feces in a tube. (**B**) Activities of fractionated fecal extracts on NF-κB reporter gene assay. C57BL/6 J (BL) and ob/ob (ob) mice feces collected at week 10 were subjected to Bligh–Dyer extraction. The aqueous and organic phases (equivalent to 10 mg feces) were exposed to murine macrophage RAW264 cells for 6 hours after being transiently transfected with pNL3.2 NF-κB RE vector. (**C**) Dose-dependent response in NF-κB reporter gene assay. Fecal extracts (equivalent to 2.5, 5, and 10 mg feces) were exposed to RAW264 cells for 6 hours. (**D**). BL and ob feces collected at week 20 were extracted and exposed to RAW264 cells for 6 hours. NF-κB reporter gene assays were performed as described in the Materials and Methods. Data are mean ± SEM (n = 3). *p < 0.05, **p < 0.01 when compared with the BL counterpart (t-test).

Fecal extracts prepared by Bligh–Dyer extraction (Fig. 3A) were applied to LC-MS non-target metabolomics. Principal component analysis (PCA) demonstrated that feces samples collected at week 0 were clearly distinguished from others (collected from weeks 6 to 22), which was due to the changeover from breeder chow (CE-2) to experimental chow (AIN-76) during week-1 (when the mice were 4 weeks old) (Fig. S1). In other words, PCA distribution of samples collected at weeks 6 to 22 seemed to be homeostatically converged in PCA score plot (Fig. S1). Since fecal metabolomics directly reflects the diet-dependent change of gut environment, continuous consumption of same diet (AIN-76) enables to eliminate possible interferes in subsequent data analysis. Therefore, feces collected at weeks 10 and 20 were subjected to NF-κB reporter gene assays to evaluate the proinflammatory activities of the fecal extracts. Murine macrophage RAW264 cells transfected with pNL3.2 NF-κB-RE vector were exposed to fecal extracts. The aqueous phase, but not the organic phase, equivalent to 10 mg feces from C57BL/6 J and *ob*/*ob* mice collected at week 10 significantly induced NF-κB activation, as determined by relative luminescence units (RLUs) (Fig. 3B). The RLUs induced by the aqueous phase of *ob*/*ob* mice was significantly higher than that of C57BL/6 J mice (Fig. 3B). These RLUs were dose-dependently increased by treatment with fecal extracts (Fig. 3C) We observed that the aqueous extract from *ob*/*ob* mice feces collected at week 20 also induced stronger NF-κB activation than that of C57BL/6 J mice (Fig. 3D). Additionally, higher levels of RLUs induced by the aqueous phase of *ob*/*ob* mice feces were also observed in human colon cancer HCT116 and HT29 cells (Fig. S2). These findings suggest that the aqueous fraction of *ob*/*ob* fecal extracts contained potential proinflammatory compounds.

### Proinflammatory activities of fractionated fecal aqueous extracts

**Figure 4:**
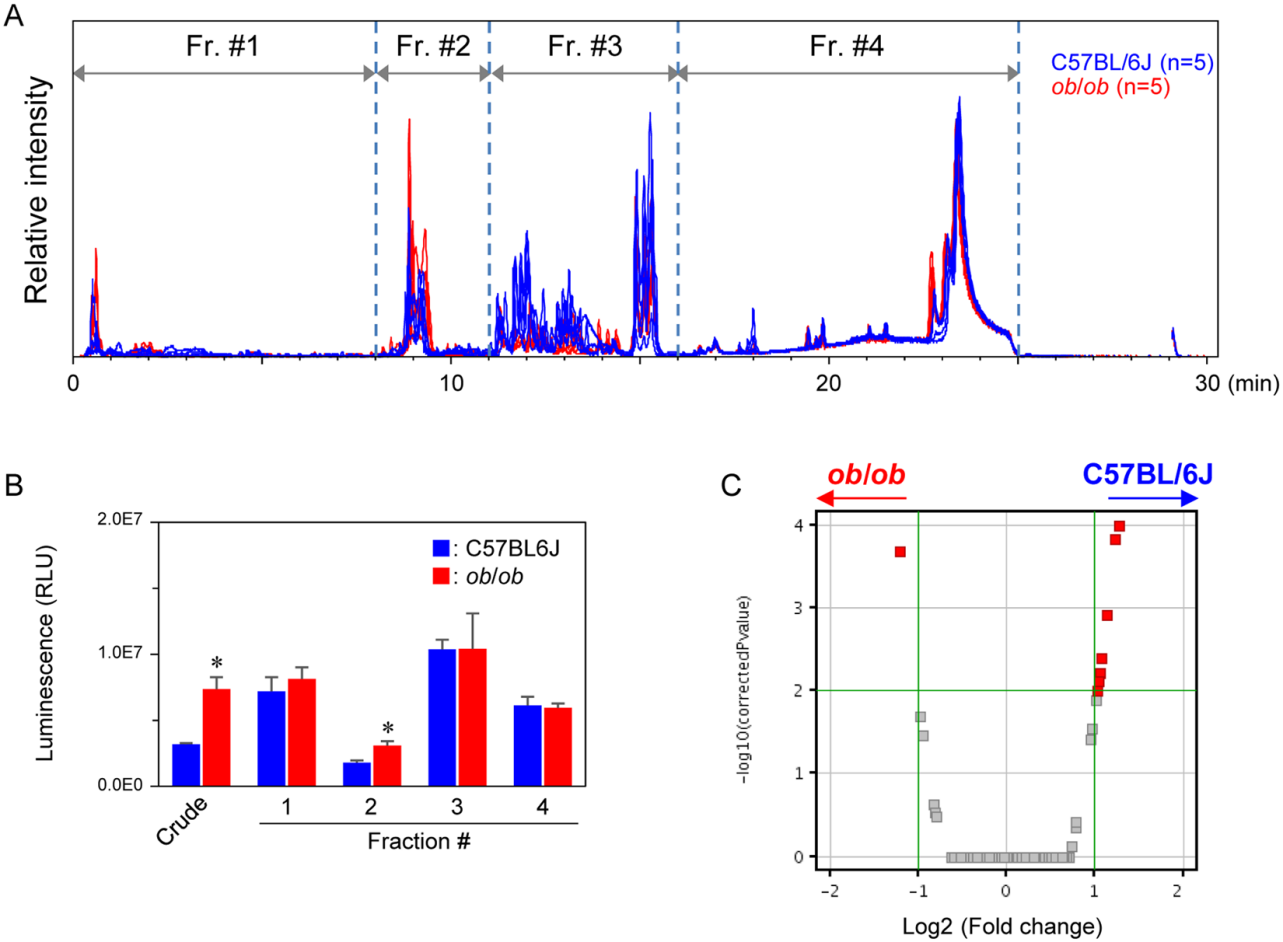
LC-MS analyses of mice fecal aqueous extracts at week 10. (**A**) Base peak ion chromatograms (ESI + ) obtained by analyses of fecal aqueous extracts. Fecal aqueous extracts injected into LC-MS were fractionated to Fr#1 to 4 as indicated. (**B**) NF-κB reporter gene assay for fractionated fecal extracts. RAW264 cells transiently transfected with pNL3.2 NF-κB RE vector were treated with crude extracts and Fr#1 to 4 for 6 hours. Data are mean ± SEM (n = 3). *p < 0.05 when compared with the BL counterpart (*t*-test). (**C**) Volcano plot analysis for fecal extracts. MS profile data were subjected to volcano plot analysis to compare the *ob/ob* and C57BL/6 J groups. A plot located in the upper left area was significantly higher in the *ob/ob* group.

**Figure 5:**
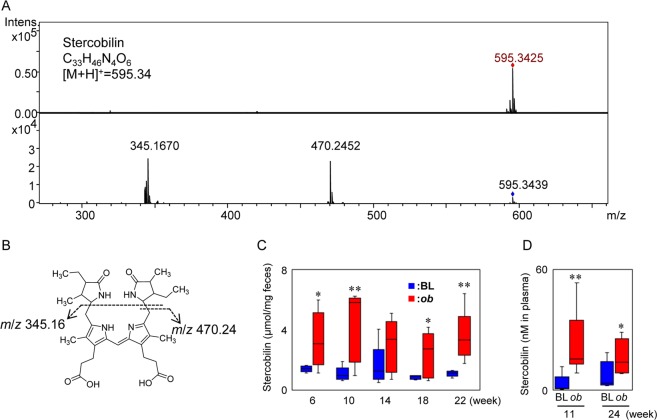
MS/MS analysis of metabolites in *ob*/*ob* mice feces. (**A**) MS and MS/MS spectra for *m/z* 595.3425 eluted at 9.0 min. The MS ion was fragmented at CE 40 eV. (**B**) Chemical structure and the collision-induced dissociation of stercobilin. (**C**) Levels of fecal stercobilin in C57BL/6 J (BL, n = 5) and *ob/ob* (*ob*, n = 5) mice. (**D**) Level of plasma stercobilin in C57BL/6 J (BL, n = 4) and *ob/ob* (*ob*, n = 4) mice. Plasma (11 and 24 weeks) extracts were analyzed in duplicate by LC-MS. Stercobilin was quantified using the external standard method. Data are shown by whisker plot. *p < 0.05, **p < 0.01 when compared with the BL counterpart (*t*-test).

Because fecal aqueous extracts of *ob/ob* mice induced strong NF-κB activities, LC-MS analysis was performed to explore the proinflammatory compounds of the extracts. As shown in Fig. 4A, chromatograms were roughly divided to four fractions. These fractions were collected and applied to NF-κB reporter gene assays. It was confirmed that NF-κB activities induced by fecal extracts (crude) from *ob*/*ob* mice were significantly higher than those of C57BL/6 J mice (Fig. 4B). Moreover, we found that fraction #2 of *ob/ob* mice eluted between 8 and 11 min significantly increased NF-κB activity compared with that of C57BL/6 J mice, although other fractions (fractions #1, 3, and 4) showed no difference in induction between the C57BL/6 J and *ob/ob* groups. A volcano plot revealed that one MS ion peak *m/z* 595.34 at 9.0 min was extracted as a characteristic MS ion peak in fraction #2 of the *ob/ob* group (Fig. 4C). To identify the compound (*m/z* 595.34 at 9.0 min), MS/MS analysis was performed. As shown in Fig. 5, we observed two prominent fragment ions, which were *m/z* 345.1670 and 470.2452 (Fig. 5A). The molecular formulas of the precursor ion (*m/z* 595.34) and fragment ions (*m/z* 345.1670 and 470.2452) were estimated as C_33_H_46_N_4_O_6_, C_19_H_24_N_2_O_4_, and C_26_H_35_N_3_O_5_, respectively. A database search was performed, then this metabolite was predicted as stercobilin. An LC chromatogram and MS and MS/MS spectra were exactly matched to the standard, thus the metabolite was assigned as a stercobilin (Fig. 5B). Although there was some individual variability within the group, the fecal levels of stercobilin in *ob/ob* mice were obviously higher than those of C57BL/6 J mice at weeks 6–22 (Fig. 5C). Although it was not significant, the plasma levels of stercobilin in *ob/ob* mice was 1.8-fold higher than those of C57BL/6 J mice (Fig. 5D). These results suggest that stercobilin is a potential biomarker for diabetic/obese model animals.

### Proinflammatory activities of stercobilin in RAW264 cells

**Figure 6:**
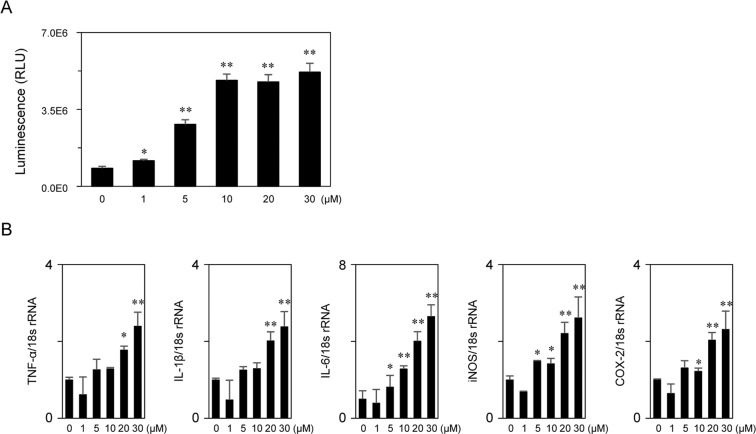
Stercobilin induces proinflammatory activities. (**A**) RAW264 cells transiently transfected with pNL3.2 NF-κB RE vector were exposed to stercobilin at the indicated concentrations for 6 hours. NF-κB reporter gene assays were performed as described in the Materials and Methods. (**B**) RAW264 cells were treated with stercobilin at the indicated concentrations for 6 hours. RT-qPCR was performed as described in the Materials and Methods. Data are mean ± SEM (n = 3). *p < 0.05 and **p < 0.01 compared with 0 µM (control) (ANOVA).

To confirm the proinflammatory activities of stercobilin, we performed NF-κB reporter gene assay using a stercobilin standard. As a result, stercobilin significantly induced NF-κB reporter gene activities in a dose-dependent manner (Fig. 6A). Additionally, stercobilin significantly induced expression of several inflammation-related genes, including TNF-α, IL-1β, IL-6, iNOS, and COX-2 (Fig. 6B). These results suggest that the higher levels of stercobilin observed in *ob/ob* mice feces contributed to the low level chronic inflammation in *ob/ob* mice.

## Discussion

In this study, to explore proinflammatory fecal metabolites in obese/diabetic model *ob/ob* mice, we performed metabolomics (LC-MS) and bioassay (NF-κB reporter gene assay). Here we showed that levels of stercobilin were higher in *ob/ob* mice feces and plasma than those of control C57BL/6 J mice. Furthermore, stercobilin induced NF-κB activities and inflammatory gene expression in culture cells. Because bioactive metabolites themselves trigger subsequent pathological events, stercobilin is a potential biomarker for diagnosing disorders at an early stage.

Stercobilin, a brown pigment in feces, is formed by catabolic conversion by intestinal bacteria. Metabolites formed by intestinal bacteria, such as urobilinogen, urobilin, stercobilinogen, and stercobilin, are collectively referred as urobilinoids^16^. Although the physiological roles of urobilinoids are not understood, the functional roles of bilirubin in the human body has been well studied. For example, in one study, bilirubin showed antioxidative activity via radical scavenging^21,22^ and in another study, bilirubin reduced adiposity and fatty liver in obese subjects via binding with PPARα^19^. It is reported that plasma levels of bilirubin are lowered in obese humans^23^ and negatively correlate with colorectal cancer in people in the United States^24^. Therefore, bilirubin within the physiologically healthy range seems to play favorable roles to prevent lifestyle-related diseases, although a pathologically elevated level of bilirubin in plasma is reflective of liver disease. However, it is unclear so far whether stercobilin has direct roles in any signaling pathway associated with obesity, diabetes or cancer. Interestingly, in one study, the fecal stercobilin level was significantly lowered (by 40%–45%) in autism spectrum disorder (ASD) model mice compared with sex matched controls^25^. Additionally, deletion of *Chd8*, a gene responsible for ASD, induced not only delayed the neurodevelopment directly associated with major ASD symptoms, but also induced slender habitus via inhibition of C/EBPβ-regulating adipogenesis^26^. These findings suggest that stercobilin is involved in obesity/diabetes mellitus through not only proinflammatory activities but also lipid metabolism in the C/EBPβ pathway. Another study used metabolomics of cecal and liver tissues to show that urobilinoids were elevated in high fat diet-induced obese C57BL/6 N mice^27^. Therefore, bilirubin formed by host metabolism and urobilinoids formed by intestinal bacteria have a similar chemical structure, but they may show opposite activities in the body. This plausible suggestion is supported by a secondary bile acid, deoxycholate, which is also formed by intestinal bacterial metabolism and is capable of inducing the cell senescence associated with liver carcinogenesis^13^. Bacterial 16 s rRNA sequencing, a powerful tool to clarify the profile of microflora, has opened new fields and markedly advanced our understanding of the interaction between the host and its bacteria. In the current study, 16 rRNA sequencing analysis revealed that the ratio of *Lachnospiraceae*, one of the bacteria of *Firmicutes*, was markedly higher in *ob/ob* mice (Table S1). Interestingly, it has been reported that intestinal colonization by a *Lachnospiraceae* contributed to the development of diabetes in germ-free *ob/ob* mice^28^. Further analysis must confirm whether *Lachnospiraceae* has a capacity to produce stercobilin. Therefore, in addition to 16s rRNA sequencing, fecal metabolomics is a crucial approach to discovering the etiology of intestinal microbiota induced pathophysiological alterations of the host.

Intestinal bacterial species, including *Bacteroides fragilis*, *Clostridium ramosum*, *Clostridium perfringens*, and *Clostridium difficile*, have been shown to catabolize bilirubin to urobilinogen^29–32^, although we could not assign these bacterial species in our results (Fig. 1C). Additionally, we detected proinflammatory stercobilin in not only feces but also plasma (Fig. 5D), suggesting the intestinal reabsorption of stercobilin. This absorbability of urobilinoids was found and reported in another study^27^. Multidrug resistant protein (MRP2) is a transporter for bilirubin diglucuronide excreted into bile and MRP3 is a transporter for bilirubin reabsorption into blood stream^33^. However, urobilinoid transporters that contribute to the enterohepatic circulation of urobilinoids have not been identified. Several mechanisms that describe how stercobilin is highly produced in *ob/ob* mice can be speculated, for example, (i) increased heme degradation by hepatopathy, (ii) increased deconjugation of bilirubin diglucuronide by microbial changes and (iii) lowered reabsorption of urobilinogen. However, detailed mechanisms are not understood yet.

The concentration of stercobilin required for the NF-κB activation in RAW264 cells were ~ 1000 times higher than those observed in *ob/ob* mice plasma. RAW264 cells required ~ μm stercobilin to activate NF-κB, which was “one shot” treatment in *in vitro*. On the other hand, in a clinical condition and *in vivo* experiments, it is well known that a low-level but chronic inflammation is strongly associated with life-style related diseases. In the current study, we detected higher levels of plasma stercobilin during an experimental period (Fig. 5D), suggesting a continuous exposure of higher levels of stercobilin to *ob/ob* mice. Moreover, in our preliminary experiment using C57BL/6 mice, treatment with 10 ppm (0.001%) urobilinogen in drinking water for 8 weeks slightly (3-fold increase) but significantly up-regulated levels of plasma urobilinogen, and inflammatory cytokines including TNF-α, IL-1β, IL-6 in liver, suggesting that urobilinoids potentially induce proinflammatory activities in *in vivo*, even if the concentration is ~nM order (unpublished observations). Therefore, we must confirm in our further study whether ~nM stercobilin in circulating blood is able to induce a chronic inflammation associated with lifestyle related disease.

Although levels of stercobilin were elevated in the feces in *ob/ob* mice, some other peaks in LC-MS were significantly higher in C57BL/6 J mice compared with *ob/ob* mice (Fig. 4C). Among them, one metabolite was identified, 5′-deoxy-5′-(methylthio)adenosine (MTA). It has been reported that MTA showed anti-oxidative activities^34^. Therefore, a lowering of antioxidative metabolites such as MTA in the intestinal tract could be another mechanism that contributes to low level chronic inflammation in lifestyle-related diseases.

In this study, we investigated intestinal metabolites soluble in aqueous and organic solvent and found that stercobilin was higher in *ob/ob* mice feces than that of controls, and that it induced moderate proinflammatory activities. Many critical metabolites have been discovered and identified using LC-MS, GC-MS, CE-MS, and NMR. In contrast, another important class in the intestinal tract are “insoluble” interphase of Bligh–Dyer extraction (Fig. 3A), which includes the strongest inflammatory molecule, bacterial LPS^35^. Low grade elevation of plasma LPS (metabolic endotoxemia) was found in high fat diet fed mice and humans^36,37^; therefore, LPS was thought to be an etiologic factor for inducing inflammation-related metabolic syndrome. Thus, further analysis might be required to determine how microbial compositional changes affect inflammatory activities of fecal and plasma LPS.

Although metabolites have been identified, the physiological activities and function of stercobilin in the mammalian body have not been examined. In addition to these metabolites, it is speculated that 3118 small-molecule biosynthetic gene clusters in genomes of human-associated bacteria have been identified^38^. Therefore, fecal metabolomic analysis can possibly find novel molecules critical in maintaining metabolic homeostasis. Further analyses are required to expand studies using other model animals and human specimens. Because the heme catabolic pathway maybe closely related to lipid metabolism, identification of the transporter for urobilinoids and regulation of bacteria producing urobilinoids by drugs or diet could be therapeutic targets and promising approaches for prevention of lifestyle-related diseases.

## Materials and Methods

### Animal experiments

All animal experiments were approved by the animal ethics committee of the University of Shizuoka (approval number 125005), and performed according to guidelines for the care and use of laboratory animals at the University of Shizuoka. Six-week-old male *ob*/*ob* (n = 5) and C57BL/6 J (n = 5) mice were purchased from CLEA Japan, Inc. (Tokyo, Japan). All animals were housed individually in plastic cages and had free access to a basal diet (AIN-76, Oriental Yeast, Co., Ltd., Tokyo, Japan) and tap water under controlled conditions of humidity (55 ± 5%), light (12/12-h light/dark cycle) and temperature (23 ± 1°C). The experimental period was for 24 weeks, following a 1-week adaptation period (Fig. 1A). During the experimental period, mice were placed into a metabolic cage, and their fecal samples were collected at indicated time points. Aliquots of collected feces were immediately stored at −80°C until use. Liver, white adipose tissues and colonic mucosa collected at week 24 were examined by RT-qPCR, hematoxylin–eosin (HE) staining, and immunohistochemistry (IHC). For IHC, frozen sections were treated with HistoVT One (Nakalai tesque, Kyoto, Japan) for of antigen retrieval, and inactivated endogenous peroxidase activity. After the blocking using Blocking One Histo (Nakalai tesque), the sections were incubated with primary antibody (Biotine anti-mouse F4/80 antibody, BioLegend, San Diego, CA). Visualization of antibody binding was performed using Streptavidin-HRP (Abcam, Cambridge, UK) and Histofine DAB Stain kit (Nichirei Corporation, Tokyo, Japan).

### NGS 16s sequencing

Microbial DNA in feces was extracted using QIAamp DNA Stool Mini Kit (QIAGEN) and the V1 and V2 variable region of 16 s rRNA was amplified using a specific primer, which consisted of target sequences (V1_F_5′-AGR GTT TGA TYM TGG CTC AG-3′, V1_R_5′-TTA CTC ACC CGT YCG CCR CT-3′, V2_F_5′-AGY GGC GRA CGG GTG AGT AA-3′ and V2_R_5′-TGC TGC CTC CCG TAG GAG T-3′), an adapter, and sample ID discriminating barcodes. The amplicon libraries prepared using the Ion PGM Sequencing 200 kit v2 PGM were loaded onto an Ion 314 chip, then sequenced using the Ion Torrent PGM. (Life Technologies, Carlsbad, CA). Data were analyzed by Ion Reporter (Life Technologies).

### RT-qPCR

Total RNA extracted using a TRIzol reagent (Invitrogen) was converted into cDNA using PrimeScript RT Master Mix (TaKaRa). To quantitatively estimate the level of each gene, quantitative PCR was performed using gene-specific primers, cDNA and SYBR Premix (TaKaRa). The sequences of the PCR primer pairs are as follows: *18 s rRNA*; 5′-CTT CTC CAT GTC GTC CCA GT-3′ and 5′-ACG CTG AGC CAG TCA GTG TA-3′, *TNF-α*; 5′-GAT TAT GGC TCA GGG TCC AA-3′ and 5′-CCC AGC ATC TTG TGT TTC TG-3′, *IL-1β*; 5′-TCT TCC TAA AAG TAT GGG CTG GA-3′ and 5′-AAA AGG GAG CTC CTT AAC ATG C-3′, *IL-6*; 5′-CGC TAT GAA GTT CCT CTC TGC-3′ and 5′-TTG GGA GTG GTA TCC TCT GTG-3′, *iNOS*; 5′-GGT ATG CTG TGT TTG GCC TTG-3′ and 5′-TTC GTC CCC TTC TCC TGT TG-3′, and *COX-2*; 5′-GGA GGC GAA GTG GGT TTT AAG-3′ and 5′-TTG ATG GTG GCT GTT TTG GTA G-3′

### Cell culture and NF-κB assay

Murine macrophage RAW264 cells (RIKEN Cell Bank, Ibaraki, Japan) and human colon cancer HCT116 (RIKEN Cell Bank, Ibaraki, Japan) and HT29 (ATCC) were maintained in 10% FBS/DMEM supplemented with 50 U/ml penicillin and 50 µg/ml streptomycin and grown in an atmosphere of 95% air and 5% CO_2_ at 37°C. NF-κB reporter gene assay was performed after the transient transfection of pNL3.2 NF-κB-RE vector (Promega) using X-tremeGENE HP DNA Transfection Reagent (Roche). Luciferase activities were evaluated using the Nano-Glo Luciferase assay kit.

### Bligh–dyer extraction of feces

Fecal extracts were prepared by the Bligh–Dyer extraction method. Briefly, 30 mg feces suspended in a tube containing 0.5 mg of *tert*-butylhydroquinone (tBHQ) and 650 µL of MeOH/CHCl_3_/H_2_O = 400/200/40 was microdestructed (3000 rpm, 4°C, 120 s ×2 sets) by MicroSmash MS-100R (TOMY, Japan) with a 5.0 φ zirconia (ZrO_2_) bead. After remove the ZrO_2_ bead, the tube was centrifuged, and the aqueous and organic phases were harvested in new tubes. Soluble metabolites were extracted again by adding 200 µL of MeOH/CHCl_3_ = 100/100 to the residue, vortexed, centrifuged, combined with former ones, then dried by a centrifuge evaporator.

### LC-MS

Fecal metabolites were analyzed using LC-MS consisting of AQUITY UPLC (Waters, Milford, MA) coupled with micrOTOFQII (Bruker Daltonics, Bremen, Germany). UPLC separation^39,40^ was performed with a CSH C18 column (1.7 μm, 100-mm × 2.1-mm i.d., Waters) at 40°C, using solvent A (0.1% formic acid in water) and solvent B (MeCN containing 0.1% formic acid). Samples were eluted from the column using a linear gradient of 1% solvent B from 0 to 3 min to 80% solvent B at 20–24 min. The flow rate of the mobile phase was 0.4 ml/min. The TOF-MS was operated in positive and negative ion mode using an electrospray ionization source. The detector conditions were as follows: capillary voltage at 4500 V, nebulizer at 1.6 bar, drying gas flow at 9 l/min, drying gas temperature 200°C, and the mass range between 80 and 2000 *m/z*. All analyses were performed using a low concentration tuning mix (Agilent Technologies, Palo Alto, CA) to calibrate mass accurately. MS peak data from UPLC-TOF-MS analyses were subjected to Compass Data Analysis (Bruker) and Signpost (Reifycs, Tokyo Japan) for peak detection and integration, and Mass Profiler Professional software (Agilent Technologies) for principal component analysis. For the analysis of plasma levels of stercobilin, plasma samples extracted by methanol were injected into LC-MS/MS consisting of an Agilent 1290 series HPLC coupled with a G6410B triple quadrupole tandem mass spectrometer. HPLC separation was performed with a ZORBAX Eclipse Plus C18 Rapid Resolution HD column (1.8 µm, 50 mm × 2.1 mm, Agilent) at 40°C, using solvent A (0.1% formic acid in water) and solvent B (MeCN containing 0.1% formic acid). Samples were eluted from the column using a linear gradient of 10% solvent B from 0 to 1 min to 80% solvent B at 7 min. The flow rate of the mobile phase was 0.4 ml/min. The MRM transition for stercobilin was monitored at *m/z* 595.4 > 345.2. In this analytical condition, the detection limit was 1 fmol/injection. Plasma levels of stercobilin were estimated by external standard methods.

### Statistical analysis

All data are presented as the mean ± SEM. All statistical analyses were performed with EZR (Saitama Medical Center, Jichi Medical University), a graphical user interface for R (The R Foundation for Statistical Computing). Statistical analyses of data were performed by a t-test or one-way ANOVA followed by Bonferroni’s post hoc test. Differences were considered significant when *p* < 0.05.

## Supplementary information

Supplementary Information.

## References

[1] Holmes E, Li JV, Marchesi JR, Nicholson JK (2012). Gut microbiota composition and activity in relation to host metabolic phenotype and disease risk. Cell Metab..

[2] Kinross J, Li JV, Muirhead LJ, Nicholson J (2014). Nutritional modulation of the metabonome: applications of metabolic phenotyping in translational nutritional research. Curr. Opin. Gastroenterol..

[3] Vijay-Kumar M (2010). Metabolic syndrome and altered gut microbiota in mice lacking Toll-like receptor 5. Science.

[4] Turnbaugh PJ (2006). An obesity-associated gut microbiome with increased capacity for energy harvest. Nature.

[5] Saji N (2019). Analysis of the relationship between the gut microbiome and dementia: a cross-sectional study conducted in Japan. Sci. Rep..

[6] Neufeld KM, Kang N, Bienenstock J, Foster JA (2011). Reduced anxiety-like behavior and central neurochemical change in germ-free mice. Neurogastroenterol. Motil..

[7] Goswami C, Iwasaki Y, Yada T (2018). Short-chain fatty acids suppress food intake by activating vagal afferent neurons. J. Nutr. Biochem..

[8] Khan MT, Nieuwdorp M, Bäckhed F (2014). Microbial modulation of insulin sensitivity. Cell Metab..

[9] De Vadder F (2014). Microbiota-generated metabolites promote metabolic benefits via gut-brain neural circuits. Cell.

[10] Chambers ES (2015). Effects of targeted delivery of propionate to the human colon on appetite regulation, body weight maintenance and adiposity in overweight adults. Gut.

[11] Tang C (2015). Loss of FFA2 and FFA3 increases insulin secretion and improves glucose tolerance in type 2 diabetes. Nat. Med..

[12] McNelis JC (2015). GPR43 Potentiates β-Cell Function in Obesity. Diabetes.

[13] Yoshimoto S (2013). Obesity-induced gut microbial metabolite promotes liver cancer through senescence secretome. Nature.

[14] Tang WHW (2013). Intestinal microbial metabolism of phosphatidylcholine and cardiovascular risk. N. Engl. J. Med..

[15] Wang Z (2011). Gut flora metabolism of phosphatidylcholine promotes cardiovascular disease. Nature.

[16] Hamoud A-R, Weaver L, Stec DE, Hinds TD (2018). Bilirubin in the Liver-Gut Signaling Axis. Trends Endocrinol. Metab..

[17] Wang X, Chowdhury JR, Chowdhury NR (2006). Bilirubin metabolism: Applied physiology. Curr. Paediatr..

[18] Roberts MS, Magnusson BM, Burczynski FJ, Weiss M (2002). Enterohepatic circulation: physiological, pharmacokinetic and clinical implications. Clin. Pharmacokinet..

[19] Stec DE (2016). Bilirubin Binding to PPARα Inhibits Lipid Accumulation. Plos One.

[20] Bixler JN (2014). Ultrasensitive detection of waste products in water using fluorescence emission cavity-enhanced spectroscopy. Proc. Natl. Acad. Sci. USA.

[21] Tomaro ML, Batlle AM, del C (2002). Bilirubin: its role in cytoprotection against oxidative stress. Int. J. Biochem. Cell Biol..

[22] Sedlak TW (2009). Bilirubin and glutathione have complementary antioxidant and cytoprotective roles. Proc. Natl. Acad. Sci. USA.

[23] Belo L (2014). Body fat percentage is a major determinant of total bilirubin independently of UGT1A1*28 polymorphism in young obese. Plos One.

[24] Zucker SD, Horn PS, Sherman KE (2004). Serum bilirubin levels in the U.S. population: gender effect and inverse correlation with colorectal cancer. Hepatology.

[25] Sekera, E. R. *et al*. Depletion of Stercobilin in Fecal Matter from a Mouse Model of Autism Spectrum Disorders. *Metabolomics* **13**, (2017). 10.1007/s11306-017-1277-9 PMC5685184 29147105

[26] Kita Y (2018). The Autism-Related Protein CHD8 Cooperates with C/EBPβ to Regulate Adipogenesis. Cell Rep..

[27] Walker A (2014). Distinct signatures of host-microbial meta-metabolome and gut microbiome in two C57BL/6 strains under high-fat diet. ISME J..

[28] Kameyama K, Itoh K (2014). Intestinal colonization by a Lachnospiraceae bacterium contributes to the development of diabetes in obese mice. Microbes Environ..

[29] Fahmy K, Gray CH, Nicholson DC (1972). The reduction of bile pigments by faecal and intestinal bacteria. Biochim. Biophys. Acta.

[30] Vítek L, Zelenka J, Zadinová M, Malina J (2005). The impact of intestinal microflora on serum bilirubin levels. J. Hepatol..

[31] Koníčková R (2012). Reduction of bilirubin ditaurate by the intestinal bacterium Clostridium perfringens. Acta Biochim. Pol..

[32] Vítek L (2006). Identification of bilirubin reduction products formed by Clostridium perfringens isolated from human neonatal fecal flora. J. Chromatogr. B. Analyt. Technol. Biomed. Life Sci..

[33] Sticova E, Jirsa M (2013). New insights in bilirubin metabolism and their clinical implications. World J. Gastroenterol..

[34] Zhang YN, Song M, Ng TB, Zhao L, Liu F (2013). Purification and characterization of antioxidant components from the fruiting bodies of Pleurotus abalonus including 9-beta-d-ribofuranosidoadenine, 5′-deoxy-5′-(methylthio)adenosine, and a triterpenoid. Environ. Toxicol. Pharmacol..

[35] Katz SS, Weinrauch Y, Munford RS, Elsbach P, Weiss J (1999). Deacylation of lipopolysaccharide in whole Escherichia coli during destruction by cellular and extracellular components of a rabbit peritoneal inflammatory exudate. J. Biol. Chem..

[36] Cani PD (2008). Changes in gut microbiota control metabolic endotoxemia-induced inflammation in high-fat diet-induced obesity and diabetes in mice. Diabetes.

[37] Harte AL (2012). High fat intake leads to acute postprandial exposure to circulating endotoxin in type 2 diabetic subjects. Diabetes Care.

[38] Donia MS (2014). A systematic analysis of biosynthetic gene clusters in the human microbiome reveals a common family of antibiotics. Cell.

[39] Miyoshi, N. *et al*. Development and application of a method for identification of isothiocyanate-targeted molecules in colon cancer cells. *Anal. Biochem*. **429**, (2012). 10.1016/j.ab.2012.07.018 22835833

[40] Hashidume T (2018). Identification of soybean peptide leginsulin variants in different cultivars and their insulin-like activities. Sci. Rep..

