# Supplementary material for: Intestinal microbial metabolite stercobilin involvement in the chronic inflammation of ob/ob mice
Source: Sci Rep. 2020 Apr 15;10:6479. doi: 10.1038/s41598-020-63627-y (PMC7160104; doi:10.1038/s41598-020-63627-y)
Supplement: Supplementary file 1 — Supplementary Information. [file 41598_2020_63627_MOESM1_ESM.pdf]

Supplementary Information of

Intestinal microbial metabolite stercobilin involvement in the chronic inflammation of *ob/ob* mice

Shunsuke Sanada[1], Takuji Suzuki[2], Akika Nagata[1], Tsutomu Hashidume[1], Yuko Yoshikawa[1,3], Noriyuki Miyoshi[1]*

[1]Graduate School of Integrated Pharmaceutical and Nutritional Sciences, University of Shizuoka, Shizuoka, Japan

[2]Food Environmental Design Course, Faculty of Education, Art and Science, Yamagata University, Yamagata, Japan

[3]School of Veterinary Medicine, Faculty of Veterinary Science, Nippon Veterinary and Life Science University, Tokyo, Japan

Sanada S, et al, Supplemental figure 1

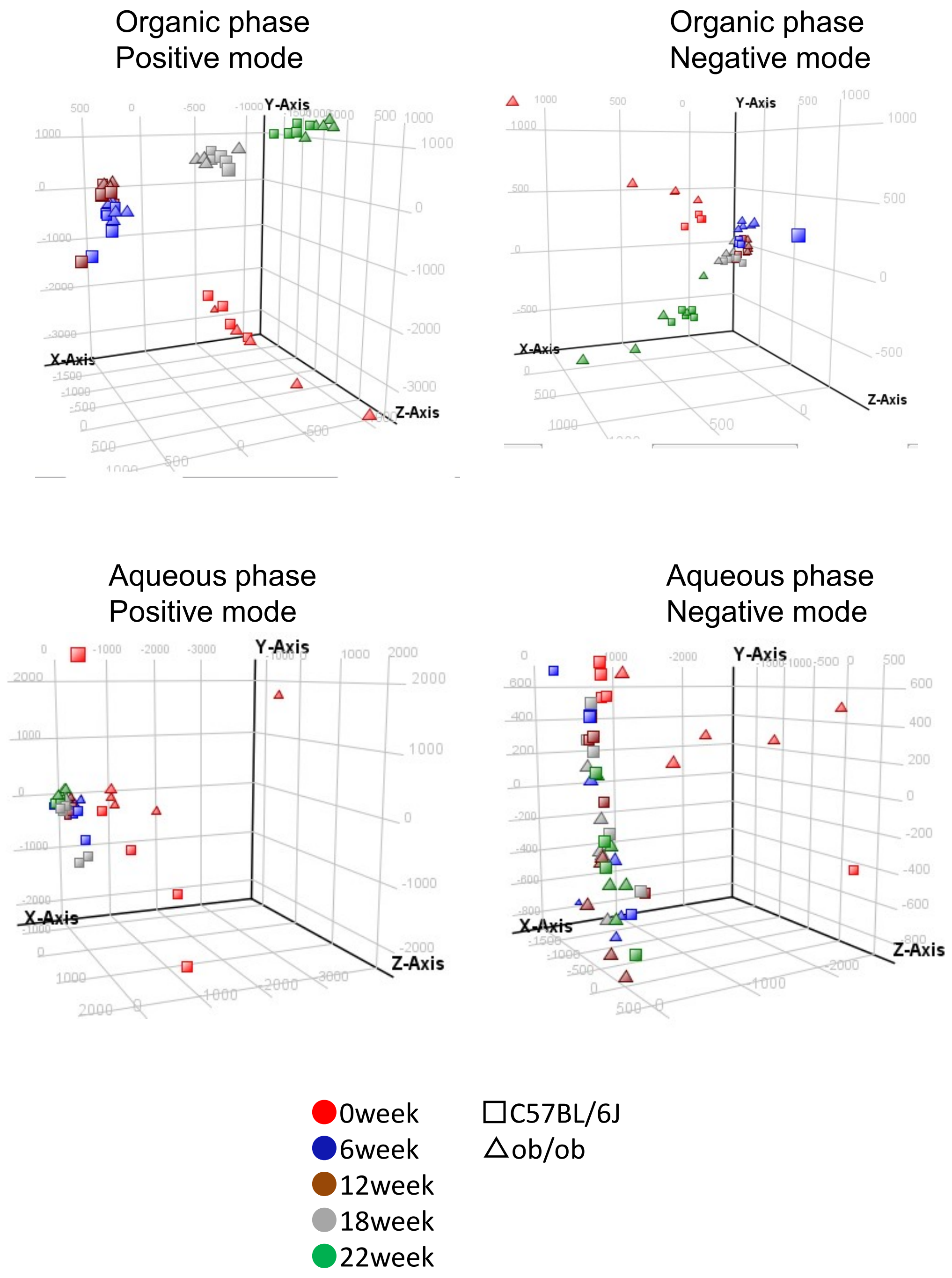

Supplemental figure 1. Principal component analysis (PCA) of fecal extracts. C57BL/6J and *ob/ob* mice feces were collected at the indicated time points. Fecal samples were subjected to Bligh–Dyer extraction to prepare the organic and aqueous extraction phases. Each extract was injected into UPLC-Q-TOF-MS. Then, raw data were processed for peak detection and integration to obtain the MS peak list. Using the MS peak list, principal component analysis (PCA) was performed by Mass Profiler Professional (Agilent Technologies, Inc.).

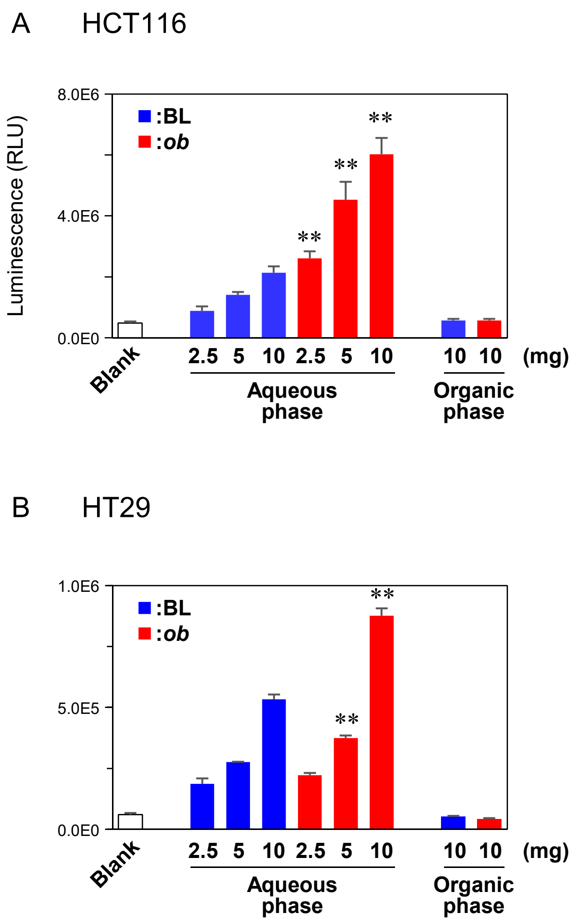

Supplemental figure 2. NF-κB reporter gene assay in human colon cancer cell lines. C57BL/6J (BL) and *ob/ob* (*ob*) mice feces collected at week 10 were subjected to Bligh–Dyer extraction. The aqueous and organic phases of fecal extracts equivalent to the indicated amounts were exposed to human colon cancer HCT116 (A) and HT29 (B) cells for 6 hours after being transiently transfected with pNL3.2 NF-κB RE vector. NF-κB reporter gene assay was performed as described in the Materials and Methods. Data are mean ± SEM (n=3). $^{**}p < 0.01$ when compared with the BL counterpart (*t*-test).

Supplemental Table 1. 16s rRNA sequencing of mice feces.
Data were represent by ratio in each samples.
*1 Data present at ≧60% in at least one condition were maked by ○.
*2 Statistical analysis were performed by 2-way ANOVA with multiple testing correction (Bonferroni family-wise error rate).

#	Phylum,Class,Order,Family,Genus,Species	Con t_06 w_1	Con t_06 w_2	Con t_06 w_3	Con t_06 w_4	Con t_06 w_5	Con t_22 w_1	Con t_22 w_2	Con t_22 w_3	Con t_22 w_4	Con t_22 w_5	Ob_ 06w_ 1	Ob_ 06w_ 2	Ob_ 06w_ 3	Ob_ 06w_ 4	Ob_ 06w_ 5	Ob_ 22w_ 1	Ob_ 22w_ 2	Ob_ 22w_ 3	Ob_ 22w_ 4	Ob_ 22w_ 5	≧60% of data in at least one condition*1	Corrected p-value (Week)*2	Corrected p-value (Week-Strain)*2	Corrected p-value (Strain)*2
1	Actinobacteria,Actinobacteria,Actinomycetales,Nocardioidaceae,(family level ID only),					0.1																			
2	Actinobacteria,Actinobacteria,Bifidobacteriales,Bifidobacteriaceae,(family level ID only),	3.2	1.9	5.2	0.1	0.5	2.2	4.3	3.5	1.8	3.1	7.3	0.1			0.7	1.8	4.6		0.1	0.3	○	1	1	1
3	Actinobacteria,Actinobacteria,Bifidobacteriales,Bifidobacteriaceae,(slash calls),	1.7	5.4	5.3	1.2	3	0.3	1.5	4.1	0.9	2	8	1.9	0.6	0.3	1.1	2.7	1.5		0.9	0.9	○	1	1	1
4	Actinobacteria,Actinobacteria,Bifidobacteriales,Bifidobacteriaceae,Bifidobacterium,(genus level ID only)	5.4	0.1	1.7	0.3	4.3	0.5	0.1	1.2		0.7	3.4	0.6	0.2	0.2					1.3	0.7	○	1	1	1
5	Actinobacteria,Actinobacteria,Bifidobacteriales,Bifidobacteriaceae,Bifidobacterium,pseudolongum	1	1	1.7	0.2	2.7	0.8	2.2	1.7	0.4	0.5	3.5	0.3		0.2		0.4	0.5		0.1		○	1	1	1
6	Actinobacteria,Actinobacteria,Coriobacteriales,Coriobacteriaceae,(family level ID only),					0.1																			
7	Actinobacteria,Coriobacteriia,Coriobacteriales,Coriobacteriaceae,(family level ID only),	0.1				0.1		0			0.1		0.1												
8	Bacteroidetes,Bacteroidia,Bacteroidales,[Odoribacteraceae],(family level ID only),			0.1				0.1								0.3				0.2					
9	Bacteroidetes,Bacteroidia,Bacteroidales,,(family level ID only),																								
10	Bacteroidetes,Bacteroidia,Bacteroidales,Bacteroidaceae,(family level ID only),				0.5	0.1	0.3					0.2	0.2	0.1	0.3	0.1					0.2	○	0.728586	1	1
11	Bacteroidetes,Bacteroidia,Bacteroidales,Bacteroidaceae,(slash calls),							0																	
12	Bacteroidetes,Bacteroidia,Bacteroidales,Bacteroidaceae,Bacteroides,(genus level ID only)	2.1	6.1	8.9	9.5	2.2	1.9	3.2	0.7	0.6	0.4	5.6	7.7	5.7	21	12	0.9	0.4		0.6	4.5	○	0.003733	1	1
13	Bacteroidetes,Bacteroidia,Bacteroidales,Bacteroidaceae,Bacteroides,(slash calls)																								
14	Bacteroidetes,Bacteroidia,Bacteroidales,Bacteroidaceae,Bacteroides,acidifaciens	0.1	0.5	0.5	2.1	0.5	0.1	1.6	0.2	0.1	0.1	0.4	1.7	1.2	0.9	2	0.2	0.8	0.3	0.8	1.3	○	1	1	1
15	Bacteroidetes,Bacteroidia,Bacteroidales,Bacteroidaceae,Bacteroides,intestinalis																								
16	Bacteroidetes,Bacteroidia,Bacteroidales,Bacteroidaceae,Bacteroides,sartorii																								
17	Bacteroidetes,Bacteroidia,Bacteroidales,Bacteroidaceae,Bacteroides,sp.	8	20	21	20	5.5	30	9.5	16	19	4.5	19	33	26	1.7		12	3.4	2.1	5.1	24	○	1	1	1
18	Bacteroidetes,Bacteroidia,Bacteroidales,Porphyromonadaceae,(family level ID only),		0.1					0.1																	
19	Bacteroidetes,Bacteroidia,Bacteroidales,Porphyromonadaceae,Parabacteroides,(genus level ID only)																								
20	Bacteroidetes,Bacteroidia,Bacteroidales,Porphyromonadaceae,Parabacteroides,distasonis				0.1			0.2						0.1											
21	Bacteroidetes,Bacteroidia,Bacteroidales,Porphyromonadaceae,Parabacteroides,goldsteinii							0.1																	
22	Bacteroidetes,Bacteroidia,Bacteroidales,Prevotellaceae,(family level ID only),																								
23	Bacteroidetes,Bacteroidia,Bacteroidales,Rikenellaceae,(family level ID only),		0.1		0.2		0.3	0.5	0.1	0.1		0.1										○	1	1	1
24	Bacteroidetes,Bacteroidia,Bacteroidales,S24-7,(family level ID only),		0.7	0.1	0.4			0.4	0.1		0.1	0.2	0.1		0.1	4.9	0.2			0.2	0.5	○	1	1	1
25	Bacteroidetes,Cytophagia,Cytophagales,Cyclobacteriaceae,(family level ID only),	0.1			0.2		0.1						0.2				0.1								
26	Bacteroidetes,Cytophagia,Cytophagales,Cytophagaceae,(family level ID only),			0.1	0.1	0.1								0.2			0.2					○	1	1	1
27	Bacteroidetes,Sphingobacteriia,Sphingobacteriales,Sphingobacteriaceae,(family level ID only),	0.1	0.1	0.1		0.1	0.1	0.1			0.1											○	1	1	0.029538
28	Cyanobacteria,Oscillatoriophycideae,Oscillatoriales,Phormidiaceae,(family level ID only),																				0.5				
29	Deferribacteres,Deferribacteres,Deferribacterales,Deferribacteraceae,Mucispirillum,schaedleri	0.4		1.7			0.1	1.2	0.1	0.1	0.8			1.8	1.3				0.4	0.1	0.8	○	0.710864	1	1
30	Firmicutes,Bacilli,Bacillales,Bacillaceae,(family level ID only),																								
31	Firmicutes,Bacilli,Bacillales,Bacillaceae,Bacillus,(genus level ID only)																								
32	Firmicutes,Bacilli,Bacillales,Bacillaceae,Bacillus,(slash calls)																								
33	Firmicutes,Bacilli,Bacillales,Staphylococcaceae,(family level ID only),																								
34	Firmicutes,Bacilli,Lactobacillales,Enterococcaceae,Enterococcus,(slash calls)																								
35	Firmicutes,Bacilli,Lactobacillales,Enterococcaceae,Enterococcus,faecalis																								
36	Firmicutes,Bacilli,Lactobacillales,Lactobacillaceae,(family level ID only),	0.3				0.4			0.1		0.2				0.2	0.4		0.1							
37	Firmicutes,Bacilli,Lactobacillales,Lactobacillaceae,(slash calls),	14	2.5	1.1	0.1	33	0.5	1.9	2.1	0.4	8.1	2.6	2	0.8	0.2	2.7	0.1	20			0.3	○	1	1	1
38	Firmicutes,Bacilli,Lactobacillales,Lactobacillaceae,Lactobacillus,(genus level ID only)					2.4					0.8			0.1			0.1	1.3							
39	Firmicutes,Bacilli,Lactobacillales,Lactobacillaceae,Lactobacillus,(slash calls)	19	2.9	8	0.7	6.2	3.6	5.6	18	4.7	17	0.1	3.2				1	7.7	0.1		2.5	○	1	1	0.386743
40	Firmicutes,Bacilli,Lactobacillales,Lactobacillaceae,Lactobacillus,acidophilus											0.1	0.1												
41	Firmicutes,Bacilli,Lactobacillales,Lactobacillaceae,Lactobacillus,animalis		0.1		0.1								0.1				0.1		0.1	0.1		○	1	1	1
42	Firmicutes,Bacilli,Lactobacillales,Lactobacillaceae,Lactobacillus,murinus																								
43	Firmicutes,Bacilli,Lactobacillales,Lactobacillaceae,Lactobacillus,reuteri																	0.9							
44	Firmicutes,Bacilli,Lactobacillales,Lactobacillaceae,Lactobacillus,sp.	0.1																							
45	Firmicutes,Bacilli,Lactobacillales,Lactobacillaceae,Lactobacillus,taiwanensis	0.4		3.9	0.3	0.4	2.1	0.1	4.5	1.5	0.3	1.1	0.1		0.3	1.6	0.5	0.3			2	○	1	1	1
46	Firmicutes,Bacilli,Lactobacillales,Streptococcaceae,(family level ID only),		0					0.1				1.3		0.2				2.5		19					
47	Firmicutes,Bacilli,Lactobacillales,Streptococcaceae,(slash calls),	0.1	1.2	0.3	0.8	0.1		0.1	2.6	0.2		0.9	0.5	0.9	2.3	0.3	2.5	8.5	1.2	2.7	0.3	○	1	1	1
48	Firmicutes,Bacilli,Lactobacillales,Streptococcaceae,Lactococcus,(genus level ID only)											0.1					0.3								
49	Firmicutes,Bacilli,Lactobacillales,Streptococcaceae,Lactococcus,(slash calls)		0.1					0.1	0.1			0.1		0.1			0.2		0.1	0.1	0.1	○	1	1	1
50	Firmicutes,Bacilli,Lactobacillales,Streptococcaceae,Lactococcus,lactis		0.1		0.1							0.1	0.1	0.1	0.1	0.1			0.2	0.2	0.1	○	1	1	1
51	Firmicutes,Bacilli,Turicibacterales,Turicibacteraceae,(family level ID only),								0.1			0.2								3.7					
52	Firmicutes,Bacilli,Turicibacterales,Turicibacteraceae,(slash calls),																			0.1					
53	Firmicutes,Bacilli,Turicibacterales,Turicibacteraceae,Turicibacter,(genus level ID only)																			0.1					
54	Firmicutes,Clostridia,Clostridiales,[Acidaminobacteraceae],(family level ID only),	0.1			0.1								0.3								0.5				
55	Firmicutes,Clostridia,Clostridiales,[Mogibacteriaceae],(family level ID only),		0.1			0.1						0.1		0.3	0.1	0.7	0.1	0.1				○	1	1	1
56	Firmicutes,Clostridia,Clostridiales,,(family level ID only),	3.9	4.3	7.8	2.2	3.7	3	2.7	3.8	5.5	4.1	4.1	2.8	5.8	2.3	6.6	7.7	6.8	11	3.1	9	○	1	1	1
57	Firmicutes,Clostridia,Clostridiales,,(slash calls),		0.7	0.1	0.2	0.1		0.3	3.2	1.4	3.3	3.5	0.1	2	3.2		8.2	1.3	5.6	1.2	5.7	○	1	1	1
58	Firmicutes,Clostridia,Clostridiales,,Clostridiales,(genus level ID only)								0.4																

59	Firmicutes,Clostridia,Clostridiales,,Clostridium,(genus level ID only)	0.2		0.3					0.1	0.2	0.1	0.1		0.1	0.4	0.1	0.2		0.1	0.7	0.2	○	1	1	1
60	Firmicutes,Clostridia,Clostridiales,,Clostridium,sp.													0.1											
61	Firmicutes,Clostridia,Clostridiales,,Lachnospiraceae,(genus level ID only)						0.7		0.5	0.3	0.7		2.2	2.5	1.2	0.5	0.1	0.1	1.5	1.3		○	1	1	1
62	Firmicutes,Clostridia,Clostridiales,,Lachnospiraceae,bacterium						0.1		0.1				0.1	0.1	0.1			0.2				○	1	1	1
63	Firmicutes,Clostridia,Clostridiales,Clostridiaceae,(family level ID only),	0.1		0.2			0.6					7.2		0.4				0.1		0.4					
64	Firmicutes,Clostridia,Clostridiales,Clostridiaceae,(slash calls),								0.2			2.3						1.7		4.9					
65	Firmicutes,Clostridia,Clostridiales,Clostridiaceae,Clostridium,(genus level ID only)											0.1				0.1		0.1							
66	Firmicutes,Clostridia,Clostridiales,Clostridiaceae,Clostridium,(slash calls)											0.1						0.2							
67	Firmicutes,Clostridia,Clostridiales,Clostridiaceae,Clostridium,aldenense																								
68	Firmicutes,Clostridia,Clostridiales,Clostridiaceae,Clostridium,disporicum								0.3			1.3					0.1	1.9		4.4		○	1	1	1
69	Firmicutes,Clostridia,Clostridiales,Eubacteriaceae,(family level ID only),	0.1											0.2	0.1			0.1								
70	Firmicutes,Clostridia,Clostridiales,Eubacteriaceae,(slash calls),																								
71	Firmicutes,Clostridia,Clostridiales,Lachnospiraceae,(family level ID only),	5.1	4.3	5.2	5.2	1.9	1.1	3.8	4.3	3.2	2.4	5.4	18	22	36	8.3	18	12	47	13	12	○	1	1	0.001462
72	Firmicutes,Clostridia,Clostridiales,Lachnospiraceae,(slash calls),	0.1			0.2	0.1		0.2			0.1		0.1	0.5	0.4	0.1	1.4		0.5	0.1	0.1	○	1	1	1
73	Firmicutes,Clostridia,Clostridiales,Lachnospiraceae,[Ruminococcus],(genus level ID only)	0.3	1.6	1.9	1.1	0.5	0.6	0.7	0.2	0.4		1.5	2.1	3.9	3.4	2.1	1	0.7	4	1	4.3	○	1	1	0.122228
74	Firmicutes,Clostridia,Clostridiales,Lachnospiraceae,[Ruminococcus],gnavus	0.3	0.4	0.4	1	0.7	0.2	0.8	0.5	0.3	0	0.6	0.7	1.2	1.7	1.7	1.3	0.7	1.8	1.1	1.1	○	1	1	0.122442
75	Firmicutes,Clostridia,Clostridiales,Lachnospiraceae,Blautia,(genus level ID only)				0.1								0.2					0.2							
76	Firmicutes,Clostridia,Clostridiales,Lachnospiraceae,Blautia,producta												0.1					0.1							
77	Firmicutes,Clostridia,Clostridiales,Lachnospiraceae,Clostridiales,(genus level ID only)	0.1	0.3	0.4	0.1	0.1		0.1			0.1											○	1	1	0.048565
78	Firmicutes,Clostridia,Clostridiales,Lachnospiraceae,Clostridiales,bacterium									0.1															
79	Firmicutes,Clostridia,Clostridiales,Lachnospiraceae,Clostridium,(genus level ID only)							0.1	0.1				0.1	0.4	0.3			0.1				○	1	1	1
80	Firmicutes,Clostridia,Clostridiales,Lachnospiraceae,Clostridium,sp.																								
81	Firmicutes,Clostridia,Clostridiales,Lachnospiraceae,Lachnospiraceae,(genus level ID only)													0.6	0.3	0.7	0.4			0.1	0.1	○	1	1	1
82	Firmicutes,Clostridia,Clostridiales,Lachnospiraceae,Lachnospiraceae,bacterium											0.1									0.2				
83	Firmicutes,Clostridia,Clostridiales,Oscillospiraceae,(family level ID only),																								
84	Firmicutes,Clostridia,Clostridiales,Peptococcaceae,(family level ID only),		0.1	0.1	0.1		0.1	0.1					0.2	0.1	0.1		0.1	0.1			0.1	○	1	1	1
85	Firmicutes,Clostridia,Clostridiales,Peptostreptococcaceae,(slash calls),											0.6			0.4			1		0.1					
86	Firmicutes,Clostridia,Clostridiales,Peptostreptococcaceae,Clostridium,irregulare																								
87	Firmicutes,Clostridia,Clostridiales,Ruminococcaceae,(family level ID only),	1.9	8.2	2.7	12	1.1	1	1.3	0.6	0.6	0.4	1.2	8.1	4.8	5.1	4.6	10	2	4.5	1.9	1.7	○	1	1	1
88	Firmicutes,Clostridia,Clostridiales,Ruminococcaceae,(slash calls),													0.2	0.1		0.2		0.2						
89	Firmicutes,Clostridia,Clostridiales,Ruminococcaceae,bacterium,(genus level ID only)													0.1											
90	Firmicutes,Clostridia,Clostridiales,Ruminococcaceae,bacterium,ASF500	0.1	0.1	0.2			0.1	0.2				0.1	0.1	0.1			0.1	0.1			0.1	○	1	1	1
91	Firmicutes,Clostridia,Clostridiales,Ruminococcaceae,Clostridiales,(genus level ID only)	0.1	0.1	0.1	0.2		0.1	0.1				0.2	0.5	0.7	0.5	0.4	0.3	0.3	0.4	0.3	0.2	○	1	1	1
92	Firmicutes,Clostridia,Clostridiales,Ruminococcaceae,Clostridiales,bacterium	0.1	0.1									0.1	0.1	0.1	0.1		0.2	0.1	0.3	0.2		○	1	1	1
93	Firmicutes,Clostridia,Clostridiales,Ruminococcaceae,Clostridium,(genus level ID only)															0.1									
94	Firmicutes,Clostridia,Clostridiales,Ruminococcaceae,Faecalibacterium,(genus level ID only)	0.8	0.8	0.1	0.2	0.8		0.1				0.5	0.2	0.8	1.7		0.2	0.4	0.2	0.1	0.6	○	1	1	1
95	Firmicutes,Clostridia,Clostridiales,Ruminococcaceae,Faecalibacterium,prausnitzii												0.1								0.1				
96	Firmicutes,Clostridia,Clostridiales,Syntrophomonadaceae,(family level ID only),																			0.1					
97	Firmicutes,Clostridia,Clostridiales,Veillonellaceae,(family level ID only),																								
98	Firmicutes,Erysipelotrichi,Erysipelotrichales,Erysipelotrichaceae,(family level ID only),	5.2	1.3	0.6	6.2	4	4.8	1.9	3.7	6.7	7.6	0.7	0.2	0.1		0.1	0.4	0.3		1.8	0.6	○	1	1	0.005783
99	Firmicutes,Erysipelotrichi,Erysipelotrichales,Erysipelotrichaceae,(slash calls),							0		0.1															
100	Firmicutes,Erysipelotrichi,Erysipelotrichales,Erysipelotrichaceae,Clostridium,(genus level ID only)	1.4	3.9	2	4.6	1.6	3.5	1.6	2.8	6.1	3.7	1.8	0.2			0.1	1.6	1.1		1.4	0.3	○	1	1	0.082371
101	Firmicutes,Erysipelotrichi,Erysipelotrichales,Erysipelotrichaceae,Clostridium,sp.				0.2	0.6		1.9		0.1	0.1		0.1									○	1	1	1
102	Firmicutes,Erysipelotrichi,Erysipelotrichales,Erysipelotrichaceae,Erysipelotrichaceae,bacterium																								
103	Firmicutes,Erysipelotrichia,Erysipelotrichales,Erysipelotrichaceae,Clostridium,cocleatum						0.1	0.3				0.3					0.1		0.1		0.4	○	1	1	1
104	Firmicutes,Erysipelotrichia,Erysipelotrichales,Erysipelotrichaceae,Turicibacter,sanguinis								0.1			0.4						0.1		1.9					
105	Proteobacteria,Alphaproteobacteria,Kiloniellales,,(family level ID only),		0.2				0.4	0.4		0.2												○	1	1	1
106	Proteobacteria,Alphaproteobacteria,Kordiimonadales,Kordiimonadaceae,(family level ID only),										0														
107	Proteobacteria,Alphaproteobacteria,Rhizobiales,Bradyrhizobiaceae,(family level ID only),																								
108	Proteobacteria,Alphaproteobacteria,Rhizobiales,Hyphomicrobiaceae,(family level ID only),																								
109	Proteobacteria,Alphaproteobacteria,Rhizobiales,Rhizobiaceae,(family level ID only),																								
110	Proteobacteria,Alphaproteobacteria,Rickettsiales,Rickettsiaceae,(family level ID only),					0.1					0.1	0.1		0.4	0.3					0.1		○	1	1	1
111	Proteobacteria,Alphaproteobacteria,Sphingomonadales,Sphingomonadaceae,(family level ID only),								0.2																
112	Proteobacteria,Alphaproteobacteria,Sphingomonadales,Sphingomonadaceae,Blastomonas,(genus level ID only)									0.1	0.2	0.1						0.1		0.1					
113	Proteobacteria,Betaproteobacteria,Burkholderiales,Alcaligenaceae,(family level ID only),	0.4	0.2	0.1		0.5	1.7	1.8		0.3	1.1	0.2	0.2	0.3			0.1				0.3	○	1	1	1
114	Proteobacteria,Deltaproteobacteria,Bdellovibrionales,Bacteriovoracaceae,(family level ID only),																								
115	Proteobacteria,Deltaproteobacteria,Bdellovibrionales,Bacteriovoracaceae,Bacteriovorax,sp.													0.1											
116	Proteobacteria,Deltaproteobacteria,Desulfovibrionales,Desulfovibrionaceae,(family level ID only),	1	20	0.2	0.9	18	28	42	1.1	4.6	3	0.5	0.4	0.2	1.2	0.1	9.8	0.2		1.1	13	○	1	1	1
117	Proteobacteria,Deltaproteobacteria,Desulfovibrionales,Desulfovibrionaceae,Desulfovibrio,(genus level ID only)	18	0.7	15	24	0.6	3	2	1.4	1.9	26	8.8	7.2	1.8	2.3	2.3	0.9	9.7	0.3	1.4	1.3	○	1	1	1
118	Proteobacteria,Deltaproteobacteria,Desulfovibrionales,Desulfovibrionaceae,Desulfovibrio,C21	0.1	0.2	0.2	0.3	0.4	0.3		16	35	0.3						0.1	0.2		12	0.1	○	1	1	1
119	Proteobacteria,Deltaproteobacteria,Desulfovibrionales,Desulfovibrionaceae,Desulfovibrio,sp.							0.1						0.1						0.3					
120	Proteobacteria,Deltaproteobacteria,Desulfuromonadales,Geobacteraceae,(family level ID only),	2.2	0.3	1.5	2.1	1.8	3.5	1.4	1.5	1.9	7.3	2.5	0.7	7	0.5		1.5	4.8	4.7		3	○	1	1	1
121	Proteobacteria,Epsilonproteobacteria,Campylobacterales,Campylobacteraceae,(slash calls),												0.3		1.9	6.3					0.1	○	1	1	1
122	Proteobacteria,Epsilonproteobacteria,Campylobacterales,Helicobacteraceae,(family level ID only),													0.1	0.4	3.5					0.4	○	1	1	1
123	Proteobacteria,Epsilonproteobacteria,Campylobacterales,Helicobacteraceae,(slash calls),											0.1		1.6	0.1	0.3			0.1	0.1	2	○	1	1	1
124	Proteobacteria,Epsilonproteobacteria,Campylobacterales,Helicobacteraceae,Flexispira,(genus level ID only)															0.1									
125	Proteobacteria,Epsilonproteobacteria,Campylobacterales,Helicobacteraceae,Helicobacter,(genus level ID only)												0.1	0.5	0.8	27				0.1	0.7	○	1	1	1
126	Proteobacteria,Epsilonproteobacteria,Campylobacterales,Helicobacteraceae,Helicobacter,(slash calls)															0.1		0.1							

127	Proteobacteria,Epsilonproteobacteria,Campylobacterales,Helicobacteraceae,Helicobacter,ganmani															5.2									
128	Proteobacteria,Epsilonproteobacteria,Campylobacterales,Helicobacteraceae,proteobacterium,(genus level ID only)																								
129	Proteobacteria,Gammaproteobacteria,Enterobacteriales,Enterobacteriaceae,(family level ID only),	0.3																							
130	Proteobacteria,Gammaproteobacteria,Enterobacteriales,Enterobacteriaceae,(slash calls),																								
131	Proteobacteria,Gammaproteobacteria,Enterobacteriales,Enterobacteriaceae,Escherichia/Shigella,coli												0.1								0.1				
132	Proteobacteria,Gammaproteobacteria,Enterobacteriales,Enterobacteriaceae,Escherichia,coli																								
133	Proteobacteria,Gammaproteobacteria,Enterobacteriales,Enterobacteriaceae,Proteus,(slash calls)																			0.1					
134	Proteobacteria,Gammaproteobacteria,Enterobacteriales,Enterobacteriaceae,Salmonella,enterica																								
135	Proteobacteria,Gammaproteobacteria,Enterobacteriales,Enterobacteriaceae,swine,manure																								
136	Proteobacteria,Gammaproteobacteria,Pseudomonadales,Pseudomonadaceae,(family level ID only),										0.1														
137	Proteobacteria,Gammaproteobacteria,Pseudomonadales,Pseudomonadaceae,(slash calls),		0.1						0.1				0.2		0.4	0.1	0.2			0.5		○	1	1	1
138	Proteobacteria,Gammaproteobacteria,Pseudomonadales,Pseudomonadaceae,Pseudomonadaceae,bacterium								0.2																
139	Proteobacteria,Gammaproteobacteria,Pseudomonadales,Pseudomonadaceae,Pseudomonas,(genus level ID or	0.1	1.2	0.7	1.2	0.1	0.6		0.4	0.3		0.2	0.3		1.8		1.9	0.3	3.4	1.6	1.3	○	1	1	1
140	Proteobacteria,Gammaproteobacteria,Pseudomonadales,Pseudomonadaceae,Pseudomonas,(slash calls)	0.8	7.6	1.2	2.5	0.7	1.4	2.1	4	0.8	0.4	0.9	1.2	1.7	3.9	1.1	10	0.6	9.9	6.5	1.3	○	1	1	1
141	Proteobacteria,Gammaproteobacteria,Pseudomonadales,Pseudomonadaceae,Pseudomonas,fragi																0.6								
142	Proteobacteria,Gammaproteobacteria,Pseudomonadales,Pseudomonadaceae,Pseudomonas,sp.		0.1			0.2		0.5			0.1	0.2	0.5	0.4	0.1	0.3				1.2	0.1	○	1	1	1
143	Proteobacteria,Gammaproteobacteria,Thiotrichales,Thiotrichaceae,(family level ID only),						0.1	0.1																	
144	Proteobacteria,Gammaproteobacteria,Xanthomonadales,Sinobacteraceae,(family level ID only),						0.7	0.3		0.2	0.1		0.4		0.1		0.1					○	1	1	1
145	Tenericutes,Mollicutes,Acholeplasmatales,Acholeplasmataceae,(family level ID only),							0.1					0.4	0.2	0.1							○	1	1	1
146	Tenericutes,Mollicutes,RF39,,(family level ID only),			0.1				0.1					0.1				0.1								
147	Verrucomicrobia,Verrucomicrobiae,Verrucomicrobiales,Verrucomicrobiaceae,(family level ID only),	0.1		0.2		0.1	0.1	0.1					0.2	0.9			0.1					○	1	1	1
148	Verrucomicrobia,Verrucomicrobiae,Verrucomicrobiales,Verrucomicrobiaceae,Akkermansia,(genus level ID only	0.3	0.2	0.3		0.1	0.1	0.1		0.1		0.1	0.3				0.1	0.1		0.1	0.3	○	1	1	1
149	Verrucomicrobia,Verrucomicrobiae,Verrucomicrobiales,Verrucomicrobiaceae,Akkermansia,muciniphila	1				0.8	2				0.2	0.1				1.1		0.1							
	Total	100	100	100	100	100	100	100	100	100	100	100	100	100	100	100	100	100	100	100	100				
